# Genetic Variants in Inflammation-Related Genes Are Associated with Radiation-Induced Toxicity Following Treatment for Non-Small Cell Lung Cancer

**DOI:** 10.1371/journal.pone.0012402

**Published:** 2010-08-25

**Authors:** Michelle A. T. Hildebrandt, Ritsuko Komaki, Zhongxing Liao, Jian Gu, Joe Y. Chang, Yuanqing Ye, Charles Lu, David J. Stewart, John D. Minna, Jack A. Roth, Scott M. Lippman, James D. Cox, Waun Ki Hong, Margaret R. Spitz, Xifeng Wu

**Affiliations:** 1 Department of Epidemiology, The University of Texas MD Anderson Cancer Center, Houston, Texas, United States of America; 2 Department of Radiation Oncology, The University of Texas MD Anderson Cancer Center, Houston, Texas, United States of America; 3 Department of Thoracic/Head and Neck Medical Oncology, The University of Texas MD Anderson Cancer Center, Houston, Texas, United States of America; 4 Hamon Center for Therapeutic Oncology Research, The University of Texas Southwestern Medical Center, Dallas, Texas, United States of America; 5 Department of Thoracic and Cardiovascular Surgery, The University of Texas MD Anderson Cancer Center, Houston, Texas, United States of America; Dresden University of Technology, Germany

## Abstract

Treatment of non-small cell lung cancer (NSCLC) with radiotherapy or chemoradiotherapy is often accompanied by the development of esophagitis and pneumonitis. Identifying patients who might be at increased risk for normal tissue toxicity would help in determination of the optimal radiation dose to avoid these events. We profiled 59 single nucleotide polymorphisms (SNPs) from 37 inflammation-related genes in 173 NSCLC patients with stage IIIA/IIIB (dry) disease who were treated with definitive radiation or chemoradiation. For esophagitis risk, nine SNPs were associated with a 1.5- to 4-fold increase in risk, including three *PTGS2* (COX2) variants: rs20417 (HR:1.93, 95% CI:1.10–3.39), rs5275 (HR:1.58, 95% CI:1.09–2.27), and rs689470 (HR:3.38, 95% CI:1.09–10.49). Significantly increased risk of pneumonitis was observed for patients with genetic variation in the proinflammatory genes *IL1A*, *IL8*, *TNF*, *TNFRSF1B*, and *MIF*. In contrast, NOS3:rs1799983 displayed a protective effect with a 45% reduction in pneumonitis risk (HR:0.55, 95% CI:0.31–0.96). Pneumonitis risk was also modulated by polymorphisms in anti-inflammatory genes, including genetic variation in *IL13*. rs20541 and rs180925 each resulted in increased risk (HR:2.95, 95% CI:1.14–7.63 and HR:3.23, 95% CI:1.03–10.18, respectively). The cumulative effect of these SNPs on risk was dose-dependent, as evidenced by a significantly increased risk of either toxicity with an increasing number of risk genotypes (*P*<0.001). These results suggest that genetic variations among inflammation pathway genes may modulate the development of radiation-induced toxicity and, ultimately, help in identifying patients who are at an increased likelihood for such events.

## Introduction

It was predicted that lung cancer would be diagnosed in over 215,000 individuals in the United States alone in 2008 [1]. Patients with locally advanced stage IIIA and IIIB (dry) disease who are not candidates for surgery are treated with definitive radiation therapy or combination chemoradiation therapy [2]. Unfortunately even with treatment, the overall 5-year survival rate for NSCLC patients is only 10–15% [3].

One of the challenges in lung cancer treatment with radiotherapy is the development of severe dose-limiting side effects. Esophagitis and pneumonitis are common acute radiation-induced normal tissue toxicities occurring in patients within one year following treatment. Presence of these toxicities can also cause a reduction in quality of life and may lead to chronic complications including lung fibrosis [4]. Currently, there are few predictors for the development of these toxicities based on clinical and dosimetric parameters [5]–[9]. Therefore, the identification of additional reliable markers could help to tailor radiation regimens in order to administer the optimal therapeutic dose while minimizing toxicity.

Inflammation is a physiological response to cellular and tissue damage, including radiation-induced damage. Appropriate response to this damage is tightly regulated through a balance between proinflammatory and anti-inflammatory cytokines and signaling molecules [10], [11]. Genetic variation in key inflammation-related genes may cause a shift in balance resulting in deregulation of the inflammatory response and corresponding modulation of susceptibility to radiation-induced normal tissue damage [12]. Previous studies have investigated genetic variation in transforming growth factor-beta 1 (TGF-β1). This important cytokine is up regulated following radiation exposure and common variants located in *TGFB1* have been found to be associated with late normal tissue complications [13]–[17].

In this study, we utilized a pathway-based approach to analyze genetic variation from 59 SNPs in 37 inflammation-related genes with regard to risk of developing either acute esophagitis or pneumonitis following radiation therapy. We explored the main effects of single SNPs and also the cumulative effect of genetic variation within the inflammation pathway on toxicity risk. These results indicate that an individual's risk of developing these severe side effects may be modulated by germline variation in inflammation genes and may help to personalize radiation therapy for NSCLC.

## Results

### Patient Characteristics

A total of 173 non-Hispanic Caucasian patients with stage IIIA (n = 70 or 40.5%) or IIIB (dry) (n = 103 or 59.5%) were included in the analysis (**Table 1**). Of these patients, 91 (52.6%) were men and 82 (47.4%) were women with a median age of 63.6 years. Most of the patients had a history of smoking with 46.8% (n = 81) being former smokers and 46.2% (n = 80) currently smoking or having quit within a year prior to diagnosis. Sixty-three (36.4%) of the tumors were classified as squamous cell carcinoma, 59 (34.1%) as adenocarcinoma, and 40 (23.1%) as non-small cell carcinoma, with the remainder (11 or 6.4%) as other NSCLC. Twenty-two patients were given a pre-treatment ECOG performance score ≥2. Nearly 80% (n = 138) of the patients received combination chemoradiation therapy, primarily with cisplatin or carboplatin (n = 142). A majority were treated with 3D radiotherapy (n = 72 or 41.6%). There were 78 occurrences of grade ≥2 esophagitis and 43 of grade ≥2 pneumonitis in our population. Twenty-three of these patients had both esophagitis and pneumonitis, while 75 patients had neither.

**Table 1:** Patient characteristics.

		Esophagitis			Pneumonitis		
	Overall	Grade <2 n(%)	Grade ≥2 n(%)	*P* value	Grade <2 n(%)	Grade ≥2 n(%)	*P v*alue
**Gender**							
Male	91	50(52.63)	41(52.56)		73(56.15)	18(41.86)	
Female	82	45(47.37)	37(47.44)	0.993	57(43.85)	25(58.14)	0.104
Total	173	95	78		130	43	
**Age, mean(SD)**	63.60(9.98)	64.85(10.14)	62.08(9.64)	0.069	64.18(10.02)	61.86(9.78)	0.188
**Smoking status**							
Never	12	6(6.32)	6(7.69)		8(6.15)	4(9.30)	
Former	81	47(49.47)	34(43.59)		53(40.77)	28(65.12)	
Current & Recent Quitter	80	42(44.21)	38(48.72)	0.733	69(53.08)	11(25.58)	**0.007**
Total	173	95	78		130	43	
**Packyr, mean(SD)**	51.62(28.67)	53.67(28.37)	49.08(29.04)	0.315	53.71(29.41)	45.07(25.48)	0.102
**Histology**							
Adenocarcinoma	59	26(27.37)	33(42.31)		42(32.31)	17(39.53)	
Squamous Cell Carcinoma	63	41(43.16)	22(28.21)		48(36.92)	15(34.88)	
Non-small Cell Carcinoma	40	21(22.11)	19(24.36)		33(25.38)	7(16.28)	
Other NSCLC	11	7(7.37)	4(5.13)	0.118	7(5.38)	4(9.30)	0.481
Total	173	95	78		130	43	
**Clinical stage**							
Stage IIIA	70	41(43.16)	29(37.18)		46(35.38)	24(55.81)	
Stage IIB(dry)	103	54(56.84)	49(62.82)	0.425	84(64.62)	19(44.19)	**0.018**
Total	173	95	78		130	43	
**Performance status**							
0	52	23(24.21)	29(37.18)		38(29.23)	14(32.56)	
1	99	59(62.11)	40(51.28)		75(57.69)	24(55.81)	
2–4	22	13(13.68)	9(11.54)	0.180	17(13.08)	5(11.63)	0.908
Total	173	95	78		130	43	
**Treatment**							
Radiation	35	31(32.63)	4(5.13)		29(22.31)	6(13.95)	
Chemoradiation	138	64(67.37)	74(94.87)	**<0.0001**	101(77.69)	37(86.05)	0.237
Total	173	95	78		130	43	
**Radiation type**							
2D	55	36(37.89)	19(24.36)		47(36.15)	8(18.60)	
3D	72	29(30.53)	43(55.13)		46(35.38)	26(60.47)	
IMRT	46	30(31.58)	16(20.51)	**0.005**	37(28.46)	9(20.93)	**0.013**
Total	173	95	78		130	43	
**Radiation dose, mean(SD)**	62.34(10.40)	60.75(12.64)	64.27(6.29)	**0.026**	61.68(11.63)	64.32(4.66)	0.149

There were no significant differences between patients who developed severe esophagitis and those who did not with regard to age, gender, smoking status, histology, clinical stage and performance status. However, patients who developed esophagitis were more likely to receive chemoradiation instead of radiation alone (*P*<0.001), and more likely to receive a higher mean radiation dose (*P* = 0.026) compared to those who did not develop esophagitis. Interestingly, severe pneumonitis was more frequent in patients who were former smokers compared to current smokers or recent quitters (*P* = 0.007). Patients with stage IIIA patients were also more likely to develop pneumonitis (*P* = 0.018). For both esophagitis and pneumonitis, there was a significant difference in the occurrence of toxicity by the type of radiation therapy administered (*P* = 0.005 and 0.013, respectively).

### Inflammation-related SNPs and Risk of Esophagitis

Among the 59 SNPs studied, a total of nine inflammation-related SNPs were found to be significantly associated with risk of esophagitis following radiation treatment (**Table 2**). All of these variants remained significant at an FDR level of 10%. In addition, because esophagitis typically presents 4–6 weeks following initiation of radiation therapy, we also analyzed the effect of these variants using logistic regression. The results are similar to those from the Cox regression analysis (data not shown).

**Table 2:** Inflammation-related SNPs and risk of esophagitis.

	Grade <2 n(%)	Grade ≥2 n(%)	*HR	95% CI	*P* value	Q value		Grade <2 n(%)	Grade ≥2 n(%)	*HR	95% CI	*P* value	Q value
***Proinflammatory cytokines, receptors, and related molecules***													
**IL6:rs1800795**	94	76					**PTGS2:rs20417**	94	76				
CC	32(34.0)	30(39.5)	1.00				GG	82(87.2)	54(71.1)	1.00			
CG	53(56.4)	27(35.5)	0.67	0.38 to 1.18	0.162		GC	**12(12.8)**	**20(26.3)**	**1.90**	**1.07 to 3.39**	**0.029**	
GG	9(9.6)	19(25.0)	1.70	0.87 to 3.35	0.123		CC	0(0.0)	2(2.6)				
CC+CG vs. GG	**85**	**57**	**2.16**	**1.18 to 3.94**	**0.013**	**0.052**	GC+CC	**12**	**22**	**1.93**	**1.10 to 3.39**	**0.029**	**0.052**
**IL16:rs11556218**	96	75					**PTGS2:rs5275**	92	75				
TT	58(60.4)	41(54.7)	1.00				TT	51(55.4)	32(42.7)	1.00			
TG	32(33.3)	22(29.3)	0.86	0.49 to 1.53	0.615		TC	39(42.4)	33(44.0)	1.43	0.85 to 2.39	0.178	
GG	**6(6.3)**	**12(16.0)**	**2.14**	**1.05 to 4.36**	**0.035**		CC	**2(2.2)**	**10(13.3)**	**2.71**	**1.25 to 5.88**	**0.011**	
TT+TG vs. GG	**90**	**63**	**2.28**	**1.16 to 4.47**	**0.017**	**0.052**	*P* for trend			**1.58**	**1.09 to 2.27**	**0.014**	**0.052**
**TNF:rs1799724**	92	71					**PTGS2:rs689470**	96	74				
CC	74(80.4)	50(70.4)	1.00				CC	92(72.3)	70(94.6)	1.00			
CT	**12(13.0)**	**20(28.2)**	**2.13**	**1.17 to 3.86**	**0.013**		CT	4(4.2)	3(4.1)	2.67	0.73 to 9.96	0.136	**0.072**
TT	6(6.5)	1(1.4)	0.91	0.12 to 6.99	0.928		TT	0(0.0)	1(1.4)				
CT+TT	**18**	**21**	**1.97**	**1.10 to 3.50**	**0.022**	**0.052**	CT+TT	**4**	**4**	**3.38**	**1.09 to 10.49**	**0.035**	
***Anti-inflammatory cytokines, receptors, and related molecules***													
**IL4R:rs1801275**	94	75					**IL10RA:rs3135932**	95	76				
AA	60(63.8)	40(53.3)	1.00				AA	68(71.6)	43(56.6)	1.00			
AG	34(36.2)	28(37.3)	0.99	0.58 to 1.68	0.973		AG	24(25.3)	28(36.8)	1.38	0.83 to 2.28	0.217	
GG	0(0.0)	7(9.3)					GG	3(3.2)	5(6.6)	2.60	0.99 to 9.83	0.053	
AA+AG vs. GG	**94**	**68**	**4.12**	**1.60 to 10.59**	**0.003**	**0.052**	*P* for trend			**1.49**	**1.01 to 2.20**	**0.046**	**0.083**
**IL10:rs1800872**	94	75											
CC	65(69.1)	43(57.3)	1.00										
CA	27(28.7)	26(34.7)	1.59	0.93 to 2.72	0.093								
AA	**2(2.1)**	**6(8.0)**	**2.88**	**1.15 to 7.22**	**0.024**								
*P* for trend			**1.65**	**1.11 to 2.45**	**0.013**	**0.052**							

*adjusted for age, gender, pack years, clinical stage, performance status, treatment regimen, radiation type, and radiation dosage.

#### Proinflammatory Genes

Of these nine SNPs, six were among genes involved in the proinflammatory response: *IL6*, *IL16*, *TNF*, and *PTGS2* (COX2). Interleukin 6 (IL6):rs1800795 resulted in an 2.16-fold increased risk (95% CI:1.18–3.94) under the recessive model. A similar effect was observed for IL16:rs11556218 (HR:2.28, 95% CI:1.16–4.47). Patients with at least one tumor necrosis factor-α (TNF) variant rs1799724 had a nearly 2-fold increased risk (HR:1.97, 95% CI:1.10–3.50). Three SNPs in *PTGS2* modulated esophagitis risk in our patient population: rs20417, rs5275, and rs689470. PTGS2:rs5275 was associated with an increased risk (*P* for trend  = 0.014). For rs20417 and rs689470, carriers of at least one variant allele were at an increased risk (HR:1.93, 95% CI:1.10–3.39 and HR:3.38, 95% CI:1.09–10.49, respectively).

#### Anti-inflammatory Genes

SNPs in the IL4 receptor, IL10, and the alpha subunit of the IL10 receptor were found to be significantly associated with increased esophagitis risk. The IL10:rs1800872 and IL10RA:rs3135932 variants were both associated with significantly increased risks under the additive model with HRs of 1.65 (95% CI: 1.11–2.45) and 1.49 (95% CI: 1.01–2.20), respectively. IL4R:rs1801275 resulted in over a 4-fold increased risk (HR:4.12, 95% CI:1.60–10.59).

#### Joint Analysis of Esophagitis Risk Alleles

**Figure 1:**
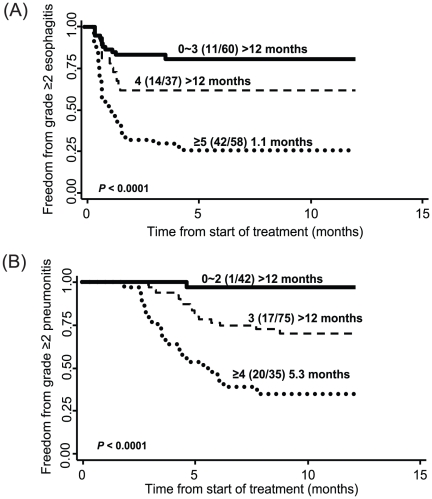
Event-free survival by number of unfavorable genotypes in inflammation-related genes. Kaplan-Meier curves of freedom from (**A**) grade >2 esophagitis or (**B**) grade >2 pneumonitis following radiation therapy. Numbers in parentheses are the number of patients with toxicity over the total number of patients; time is median event-free duration in months.

To understand the cumulative effect of unfavorable genotypes on risk of esophagitis, we performed a combined analysis. We included all significant SNPs identified from our individual SNP analysis and an additional seven SNPs reaching borderline significance at p<0.10 (**Table 3**). Patients with four unfavorable genotypes had a 3.71-fold increased risk (95% CI:1.53–8.99) compared to those with 0∼3 unfavorable genotypes. This risk increased to 8.85 (95% CI:4.19–18.68) for patients with five or more unfavorable genotypes. Furthermore, patients with an increasing number of unfavorable genotypes developed esophagitis significantly quicker following initiation of radiation therapy (**Figure 1A**). Carriers of five or more unfavorable genotypes had a median time to event of only 1.1 months compared to over 12 months for those with three or less unfavorable genotypes (*P*<0.0001).

**Table 3:** Cumulative effect of unfavorable genotypes and radiation-induced toxicity risk.

Number of Unfavorable Genotypes	Grade <2 n	Grade ≥2 n	*HR	95% CI	*P* value
**^#^Esophagitis**					
0∼3	49	11	1.00		
4	23	14	**3.71**	**1.53 to 8.99**	**0.004**
≥5	16	42	**8.85**	**4.19 to 18.68**	**<0.0001**
*P* trend					**<0.0001**
& **Phenumonitis**					
0∼2	41	1	1.00		
3	58	17	**13.30**	**1.72 to 102.94**	**0.013**
≥4	15	20	**69.42**	**8.62 to 558.91**	**<0.0001**
*P* trend					**<0.0001**

*adjusted for age, gender, pack years, clinical stage, performance status, treatment regimen,

radiation type, and radiation dosage**.**

^#^unfavorable genotypes: IL6:rs1800795, IL16:rs11556218, TNF:rs1799724, PTGS2:rs20417.

PTGS2:rs5275, PTGS2:rs689470, IL4R:rs1801275, IL10:rs1800872, IL10RA:rs3135932.

IL1B:rs16944, IL2RB:rs228942, IL8:rs4073, IL10RB:rs2834167, IL13:rs1800925, NOS2:rs2297518.

& unfavorable genotypes: IL1A:rs1800587, IL8:rs4073, TNF:rs1799724, TNFRSF1B:rs1061622.

MIF:rs7555622, IL4:rs2243250, IL4R:rs2070874, IL13:rs10800925, IL13:rs20541, NOS3:rs1799983, NFKBIA:rs1799983.

### Inflammation-related SNPs and Risk of Pneumonitis

A different set of inflammation-related SNPs was found to be significantly associated with risk of developing pneumonitis following radiation therapy and remained so at an FDR of 10% (**Table 4**). Only one of the 12 SNPs identified were also associated with esophagitis risk – TNF:rs1799724. Patients homozygous for this variant exhibited a 5.96-fold increased risk (95% CI:1.33–18.57) of pneumonitis. This risk is similar for esophagitis risk in patients carrying at least one variant allele (**Table 2**).

**Table 4:** Inflammation-related SNPs and risk of pneumonitis.

	Grade <2 n(%)	Grade ≥2 n(%)	*HR	95% CI	*P* value	Q value		Grade <2 n(%)	Grade ≥2 n(%)	*HR	95% CI	*P* value	Q value
***Proinflammatory cytokines, receptors, and related molecules***													
**IL1A:rs1800587**	128	43					**TNFRSF1B:rs1061622**	126	42				
CC	65(50.8)	11(25.6)	1.00				TT	72(57.1)	17(40.5)	1.00			
CT	**51(39.8)**	**30(69.8)**	**3.66**	**1.66 to 8.07**	**0.001**		TG	50(39.7)	22(52.4)	1.84	0.90 to 3.79	0.096	
TT	12(9.4)	2(4.7)	0.89	0.19 to 4.23	0.885		GG	**4(3.2)**	**3(7.1)**	**5.88**	**1.50 to 23.09**	**0.011**	
CT+TT	**63**	**32**	**2.90**	**1.34 to 6.25**	**0.007**	**0.021**	*P* for trend			**2.12**	**1.18 to 3.79**	**0.012**	**0.023**
**IL1A:rs17561**	128	43					**MIF:rs755622**	126	43				
GG	65(50.8)	12(27.9)	1.00				CC	89(70.6)	26(60.5)	1.00			
GT	**52(40.6)**	**29(67.4)**	**3.11**	**1.44 to 6.72**	**0.004**		CG	32(25.4)	12(27.9)	1.49	0.69 to 3.24	0.312	
TT	11(8.6)	2(4.7)	0.85	0.18 to 4.01	0.836		GG	**5(4.0)**	**5(11.6)**	**4.49**	**1.14 to 17.66**	**0.031**	
GT+TT	**63**	**31**	**2.51**	**1.19 to 5.27**	**0.015**	**0.024**	CC+CG vs. GG	**121**	**38**	**3.96**	**1.04 to 15.12**	**0.044**	**0.038**
**IL8:rs4073**	128	41					**NOS3:rs1799983**	129	42				
TT	37(28.9)	7(17.1)	1.00				GG	51(39.5)	22(52.4)	1.00			
TA	66(51.6)	19(46.3)	1.35	0.51 to 3.56	0.548		GT	61(47.3)	17(40.5)	0.53	0.27 to 1.04	0.067	
AA	**25(19.5)**	**15(36.6)**	**3.88**	**1.42 to 10.62**	**0.008**		TT	17(13.2)	3(7.1)	0.34	0.08 to 1.51	0.157	
TT+TA vs. AA	**103**	**26**	**3.16**	**1.54 to 6.48**	**0.002**	**0.010**	*P* for trend			**0.55**	**0.31 to 0.96**	**0.037**	**0.038**
**TNF:rs1799724**	121	42											
CC	94(77.7)	30(71.4)	1.00										
CT	23(19.0)	9(21.4)	1.45	0.64 to 3.26	0.370								
TT	**4(3.3)**	**3(7.1)**	**5.32**	**1.40 to 20.22**	**0.014**								
CC+CT vs. TT	**117**	**39**	**4.96**	**1.33 to 18.57**	**0.017**	**0.024**							
***Anti-inflammatory cytokines, receptors, and related molecules***													
**IL4:rs2243250**	128	42					**IL13:rs20541**	129	43				
CC	103(80.5)	27(64.3)	1.00				CC	88(68.2)	28(65.1)	1.00			
CT	**22(17.2)**	**13(31.0)**	**2.50**	**1.22 to 5.11**	**0.012**		CT	35(27.1)	9(20.9)	0.98	0.43 to 2.25	0.968	
TT	3(2.3)	2(4.8)	3.10	0.34 to 28.02	0.313		TT	**6(4.7)**	**6(14.0)**	**2.94**	**1.12 to 7.73**	**0.028**	
CT+TT	**25**	**15**	**2.54**	**1.27 to 5.08**	**0.008**	**0.021**	CC+CT vs. TT	**123**	**37**	**2.95**	**1.14 to 7.63**	**0.025**	**0.031**
**IL4:rs2070874**	128	43					**IL13:rs180925**	129	43				
CC	104(81.3)	27(62.8)	1.00				CC	83(64.3)	26(60.5)	1.00			
CT	**21(16.4)**	**15(34.9)**	**3.09**	**1.49 to 6.44**	**0.003**		CT	42(32.6)	13(30.2)	0.71	0.33 to 1.52	0.380	
TT	3(2.3)	1(2.3)	2.59	0.27 to 24.47	0.405		TT	4(3.1)	4(9.3)	2.97	0.93 to 9.45	0.066	
CT+TT	**24**	**16**	**3.05**	**1.50 to 6.22**	**0.002**	**0.010**	CC+CT vs. TT	**125**	**39**	**3.23**	**1.03 to 10.18**	**0.045**	**0.038**
**NFKBIA:rs8904**	127	43											
CC	54(42.5)	12(27.9)	1.00										
CT	53(41.7)	16(37.2)	0.99	0.42 to 2.30	0.974								
TT	20(15.7)	15(34.9)	2.00	0.84 to 4.79	0.119								
CC+CT vs. TT	**107**	**28**	**2.02**	**1.01 to 4.03**	**0.047**								

*adjusted for age, gender, pack years, clinical stage, performance status, treatment regimen, radiation type, and radiation dosage.

#### Proinflammatory Genes

Other significant genetic variants associated with pneumonitis included six SNPs in proinflammatory genes, including *IL1A*, *IL8*, *TNFRSF1B, MIF*, and *NOS3*. Two SNPs in *IL1A* – rs1800587 and rs17561– are in strong linkage disequilibrium and each resulted in a more than doubling of risk with HRs of 2.90 (95% CI:1.34–6.25) and 2.51 (95% CI:1.19–5.27), respectively. The risk associated with IL8:rs4073 was similar at 3.16-fold (95% CI:1.54–6.48). Under the additive model, TNFRSF1B:rs1061622 resulted in a 2.12-fold increased risk (95% CI:1.18–3.79). A SNP in the lymphokine gene *MIF* resulted in an even higher HR of 3.96 (95% CI:1.04–15.12). In contrast, genetic variation in *NOS3* was associated with a 50% decrease in pneumonitis risk (HR:0.55, 95% CI:0.31–0.96). This was the only SNP in our analysis to be significantly associated with a reduction in risk.

#### Anti-inflammatory Genes

IL4 and IL13 share a common receptor and have many of the same anti-inflammatory functions. In our population, we found that genetic variations in both of these interleukins were associated with increased risks of developing pneumonitis. The two *IL4* SNPs each resulted in increased risk with HRs of 2.54 (95% CI:1.27–5.08) and 3.05 (95% CI:1.50–6.22), respectively. *IL13* polymorphisms had a similar effect on pneumonitis risk. Patients with two variant alleles or either rs20541 or rs180925 were approximately 3-times more likely to develop pneumonitis compared to those with wild-type or heterozygous genotypes (HR:2.95, 95% CI:1.14–7.63 and HR:3.23, 95% CI:1.03–10.18). The signaling molecule IkappaB-alpha (*NFKBIA*) inhibits the inflammatory response by blocking NFkappaB-mediated transcription of proinflammatory genes. NFKBIA:rs8904 resulted in a 2.02-fold increased pneumonitis risk (95% CI:1.01–4.03).

#### Joint Analysis of Pneumonitis Risk Alleles

In combined analysis, the significant SNPs together with an additional borderline significant variant – IL4R: rs1801275 (*P* = 0.053) – showed an increase in pneumonitis risk as the number of unfavorable genotypes increased (**Table 3**). The increased risk for carrying three unfavorable genotypes was 13.30-fold compared to patients with 0 to 2 risk genotypes (*P* = 0.013). This risk was dramatically increased for the group of patients with four or more unfavorable genotypes (*P*<0.0001). These high risk individuals also had a shorter duration between start of treatment and development of pneumonitis of only 5.33 months compared to over 12 months for those with 0 to 2 unfavorable genotypes (**Figure 1B**).

### Inflammation-related SNPs and Overall Survival

**Figure 2:**
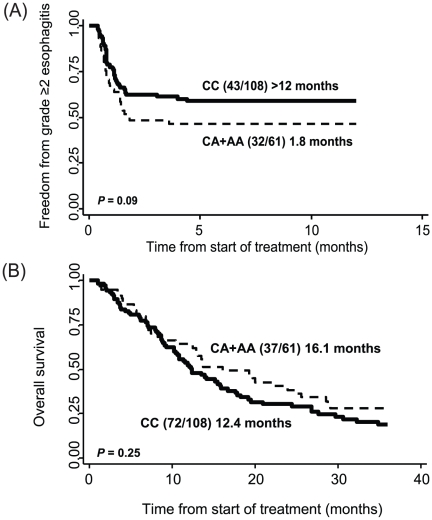
Relationship between esophagitis and overall survival. (**A**) Kaplan-Meier curves of freedom from grade >2 esophagitis following radiation therapy by IL10:rs1800872 genotypes. Numbers in parentheses are the number of patients with toxicity over the total number of patients; time is median event-free duration in months. (**B**) Kaplan-Meier curves of overall survival following radiation therapy by IL10:rs1800872 genotypes. Numbers in parentheses are the number of patients alive over the total number of patients; time is median survival time in months.

The development of toxicity and survival are often related since patients who develop toxicity are those who are responding to treatment. Therefore, we determined if any of the variants identified as toxicity risk factors were also associated with survival over three years. We found that patients with at least one variant allele of IL10:rs1800872 had a 1.74-fold increased risk of esophagitis, but a 40% decreased risk of dying when compared to patients with wild-type genotypes (HR:0.62, 95% CI:0.40–0.97). **Figure 2A** illustrates the time to esophagitis for patients with IL10:rs1800872 genotypes. Although not significant, patients with wild-type genotypes had median time to event of greater than 12 months contrasted with only 1.8 months for those with at least one variant of rs1800872. For survival (**Figure 2B**), there was a non-significant survival advantage of nearly four months for carriers with a median survival time of 16.1 months compared to only 12.4 months for patients with wild-type genotypes.

## Discussion

In this study, we systematically analyzed 59 common genetic variations in inflammation-related genes for association with risk of developing acute esophagitis or pneumonitis following radiation treatment in NSCLC patients. Multiple individual SNPs in important pro- and anti-inflammatory genes were identified as modulating risk for both normal tissue toxicities. Furthermore, the cumulative effect of these SNPs was dose-dependent with individuals carrying multiple unfavorable alleles having a corresponding increase in risk.

Nine genetic variants were identified as significantly associated with esophagitis risk, and of those, six were in proinflammatory genes (**Table 2**). We found that rs1800795 in *IL6* resulted in a 2.16-fold increase in esophagitis. This polymorphism is located within the 5′-untranslated region of *IL6* and has been functionally studied with conflicting results of the effect on gene expression and response to stimulation [18], [19]. However, a recent meta-analysis of over 5,500 patients was not able to demonstrate a relationship between this variant and IL6 serum levels [20]. Gao et al. demonstrated that IL16:rs11556218 was significantly associated with colorectal and gastric cancer, but did not observe a correlation between IL16 serum levels measured in these patients and rs11556218 [21]. PTGS2:rs20417 was also associated with increased risk of esophagitis. This promoter variant disrupts a Sp1/Sp3 transcription factor binding site and causes a decrease in transcriptional activity in lung fibroblast cells [22], [23]. Decreased expression of COX2 would suggest a decrease in inflammation signaling. However, this same variant, while altering the Sp1/Sp3 site, also introduces a binding site for another transcription factor, Egr-1, although the consequences are unknown [22]. The other two significant variants (rs5275 and rs689470) are located in the 3′-UTR and regulate *PSTGS2* mRNA levels. Our results suggest that these SNPs are linked with an increase in pro-inflammatory activity leading to esophagitis. Further functional analysis is warranted to understand the underlying mechanisms [24].

For anti-inflammatory molecules and esophagitis risk, Khurana Hershey et al. demonstrated that IL4R:rs1801275 resulted in enhanced IL4 signaling and the induction of high levels of the IgE receptor CD23 [25]. IL10:rs1800872 and IL10RA:rs3135932 have been shown to decrease IL10 signaling by decreasing serum levels and altering IL10-IL10RA interactions, respectively [26], [27]. The reported functions of these three SNPs would be in agreement with our findings of an increased risk of esophagitis by decreasing the anti-inflammatory response.

Twelve common polymorphisms were found to be significantly associated with risk of pneumonitis. The two *IL1A* variants are in linkage disequilibrium and were found to increase risk by nearly 3-fold. IL1A:rs1800587 has been shown to contribute to an increase in IL1-α promoter activity, mRNA levels and protein levels [28]. IL1A:rs17561 is a non-synonymous SNP and increases processing of the IL1-α precursor resulting in an increase in the levels of active IL1-α [29]. The variant of IL8:rs4073, which was found to increase pneumonitis risk 3-fold, has been associated with increased secretion of the proinflammatory cytokine IL8 [30]. IL4 and IL13 work together to regulate the inflammatory response. Four genetic variants in these two genes were associated with ∼3-fold increased risk of pneumonitis. Studies have demonstrated increased IgE production for IL4:rs2070874 and rs2243250 [31] and increased IL13 activity for IL13:rs20541 and rs180925 [32], [33].

Genetic variation in *TNF* and the receptor *TNFRSF1B* were also associated with increased risk of pneumonitis. TNF-α signaling is an important modulator of the inflammatory response. The TNF:rs1799724 variant is located within the promoter region of the gene and thought to influence gene expression by creating an OCT-1 transcription factor binding site [34]. The effect of this differential binding on TNF-α signaling is not clear. Some studies have shown an increase in TNF-α production [35], [36], [37], while others have shown the opposite effect [38], [39], [40]. For TNFRSF1B, the non-synonymous variant Met196Arg (rs1061622) does not alter TNF-α binding affinity, but results in intensified TNF-α signaling [41] and decreased NF-kB signaling [42].

Only one genetic variant was found to confer a protective effect following radiotherapy. This variant, rs1799983, in *NOS3* was associated with a 70% reduction in risk of pneumonitis. Functional studies have demonstrated that this variant results in production of a variant allozyme with reduced enzyme activity [43] resulting in a reduction in nitric oxide production [44]. These observations support our findings of decreased pneumonitis due to decreased inflammatory signaling.

In all, the functional consequences of the variants identified as strongly associated with increased risk of normal tissue toxicity following radiation exposure suggest a high biological plausibility for our findings. However, little to no information is known about how these variants specifically alter pneumonitis and esophagitis risk. The inflammatory response is complex and many prototypic “proinflammatory” molecules have anti-inflammatory attributes under specific conditions, and vice versa. Further studies are warranted to elucidate the specific function of these SNPs in target tissues following exposure to radiation.

Interestingly, we observed several variants with a trend towards a relationship between toxicity and overall survival, and only one SNP was identified as being associated with both. This result suggests that these patients who are developing acute normal tissue toxicity are responding well to therapy with longer survival times. Unfortunately, these side effects are dose limiting and often result in cessation of treatment. It may be that these select patients would receive the most benefit from the inclusion of radioprotective agents such as amifostine and glutamine in their treatment regimen. Both work by decreasing the levels of reactive oxygen species in the exposed normal tissue and, thus, potentially avoiding the development of inflammation. It would be of interest to test the significant SNPs identified in this study within the framework of these agents.

Our study has several advantages, including the patient population with availability of comprehensive clinical and epidemiological information. To our knowledge, no study has systematically investigated the effect of genetic variations within inflammation-related genes and risk of normal tissue toxicity due to radiation therapy. This pathway-based approach allowed us to comprehensively elucidate the cumulative effects of multiple adverse alleles on toxicity risk. Since a patient's genome can contain several of these risk associated genetic variants in both proinflammatory and anti-inflammatory pathways, this approach is much more powerful in detecting the effect of these SNPs on a patient's risk of developing esophagitis or pneumonitis. The variants included in this study were candidate SNPs based on known or predicted effects on gene function. A candidate-gene approach has the advantage of being anchored by known biological plausibility, but there is a possibility that this study has missed additional risk alleles or detected a variant in linkage disequilibrium with the true causative SNP. In addition, we were not able to include additional variables that may also impact toxicity, including radiation field size, dose to organ at risk (esophagus and lung), treatment volume, and tumor location.

In conclusion, we identified several biologically plausible associations between genetic variants in important inflammation-related genes and risk of developing esophagitis and pneumonitis. We also demonstrated a dose-effect of inflammation SNPs as evidenced by the dramatic increases in risk with increases in number of unfavorable genotypes. Furthermore, we identified one variant in IL10 that is associated with increased risk of esophagitis, but a decreased risk of dying. Since radiotherapy is a mainstay of lung cancer treatment, having the ability to screen patients prior to initiation of treatment would potentially minimize these acute toxicity events while allowing for higher doses of radiation for those who are not at increased risk in order to improve local control. With validation, these results, together with clinical and dosimetric predictors, could serve to increase the overall benefit of radiation therapy in NSCLC patients.

## Methods

### Ethics Statement

Participants gave written informed consent and the study was approved by The University of Texas MD Anderson Cancer Center's Institutional Review Board.

### Patient Population

The study included non-Hispanic Caucasian subjects who were newly diagnosed, histologically confirmed stage IIIA or IIIB without a malignant effusion (dry) NSCLC patients receiving definitive thoracic radiation or chemoradiation therapy at The University of Texas MD Anderson Cancer Center. All of the patients were enrolled in an ongoing epidemiology lung cancer study between 1995 and 2007.

### Epidemiological and Clinical Data Collection

Epidemiologic data were collected during an in-person interview using a structured questionnaire to determine demographic characteristics, medical history, and smoking history. Clinical and follow-up information was abstracted from medical records. Pre-treatment performance status was determined based on the Eastern Cooperative Oncology Group scale. Radiation-induced esophagitis was characterized by documentation of new-onset pain on swallowing occurring during treatment. Pneumonitis was detected by roentgenographic or CT scan abnormalities and often associated with nonproductive cough and/or fever. Severity of pneumonitis or esophagitis was scored by the physician according to National Cancer Institute Common Terminology Criteria for Adverse Events (version 3.0) guidelines [45]. For this study, an event was considered the occurrence of grade ≥2 toxicity.

### SNP Selection and Genotyping

Blood was drawn from each participant following the in-person interview. These samples were used to extract genomic DNA from peripheral blood lymphocytes using the Human Whole Blood Genomic DNA Extraction Kit (Qiagen, Valencia, CA). A total of 59 candidate SNPs (**Table 5**) were selected from 37 known inflammation-related genes as previously described [46]. Briefly, candidate SNPs were selected if they had a minor allele frequency greater than 5% and were located in a putative functional region of the gene (promoter, untranslated regions (UTR) or exons) or had previously been reported as associated with cancer or an inflammatory disorder. Genotyping was performed using the SNPlex assay following manufacturer's instructions (Applied Biosystems, Foster City, CA) with analysis on an Applied Biosystems 3730 DNA Analyzer. SNP genotypes were called using the GeneMapper software (Applied Biosystems). Three SNPs: IL8RA:rs2234671, LTA:rs2229092 and IL4R:rs1805011 were removed because of excessive missing genotypes (>20%). All genotyping was completed blinded with regard to toxicity status.

**Table5:** Inflammation single nucleotide polymorphism characteristics.

dbSNP ID	Alleles	Gene Symbol	Gene Name	SNP Location*
rs1800872	C/A	IL10	interleukin 10	5'-FR
rs1800896	G/A	IL10	interleukin 10	5'-FR
rs1900871	A/C	IL10	interleukin 10	5'-FR
rs3135932	A/G	IL10RA	interleukin 10 receptor, alpha	Ser159Gly
rs2834167	A/G	IL10RB	interleukin 10 receptor, beta	Lys47Glu
rs1800925	C/T	IL13	interleukin 13	5'-FR
rs20541	C/T	IL13	interleukin 13	Arg130Gln
rs2070874	C/T	IL4	interleukin 4	5'-UTR
rs2243250	C/T	IL4	interleukin 4	5'-FR
rs1801275	A/G	IL4R	interleukin 4 receptor	Gln576Arg
rs1805010	A/G	IL4R	interleukin 4 receptor	Ile75Val
rs1805011	A/C	IL4R	interleukin 4 receptor	Glu400Ala
rs1805015	T/C	IL4R	interleukin 4 receptor	Ser503Pro
rs1805016	T/G	IL4R	interleukin 4 receptor	Ser752Ala
rs2069812	C/T	IL5	interleukin 5 receptor	5'-FR
rs2233409	C/T	NFKBIA	IkB alpha	5'-FR
rs8904	C/T	NFKBIA	IkB alpha	3'-UTR
rs1800206	C/G	PPARA	peroxisome proliferator-activated receptor alpha	Leu162Val
rs2016520	A/G	PPARD	peroxisome proliferator-activated receptor delta	5'-UTR
rs1801282	C/G	PPARG	peroxisome proliferator-activated receptor gamma	Pro12Ala
rs1024611	T/C	CCL2	chemokine (C-C motif) ligand 2	5'-FR
rs2069614	C/T	CSF2	colony stimulating factor 2 (granulocyte-macrophage)	5'-FR
rs25882	T/C	CSF2	colony stimulating factor 2 (granulocyte-macrophage)	Ile117Thr
rs2257167	G/C	IFNAR1	interferon (alpha, beta and omega) receptor 1	Val168Leu
rs1051393	T/G	IFNAR2	interferon (alpha, beta and omega) receptor 2	Phe10Val
rs2069705	T/C	IFNG	interferon, gamma	5'-FR
rs2430561	A/T	IFNG	interferon, gamma	intron
rs3212227	A/C	IL12B	interleukin 12B	3'-UTR
rs375947	A/G	IL12RB	interleukin 12 receptor, beta 1	Met365Thr
rs11556218	T/G	IL16	interleukin 16	Asn446Lys
rs4778889	T/C	IL16	interleukin 16	5'-FR
rs17561	G/T	IL1A	interleukin 1, alpha	Ala114Ser
rs1800587	C/T	IL1A	interleukin 1, alpha	5'-FR
rs1143627	T/C	IL1B	interleukin 1, beta	5'-FR
rs1143634	C/T	IL1B	interleukin 1, beta	Phe105Phe
rs16944	C/T	IL1B	interleukin 1, beta	5'-FR
rs2228139	C/G	IL1R1	interleukin 1 receptor, type I	Ala124Gly
rs2069762	T/G	IL2	interleukin 2	5'-FR
rs228942	C/A	IL2RB	interleukin 2 receptor, beta	Asp391Glu
rs1800795	C/G	IL6	interleukin 6 (interferon, beta 2)	5'-FR
rs2228145	A/C	IL6R	interleukin 6 receptor	Asp358Ala
rs4073	T/A	IL8	interleukin 8	5'-FR
rs2234671	G/C	IL8RA	interleukin 8 receptor, alpha	Ser276Thr
rs2229092	A/C	LTA	lymphotoxin alpha	His51Pro
rs2229094	T/C	LTA	lymphotoxin alpha	Arg13Cys
rs755622	C/G	MIF	macrophage migration inhibitory factor	5'-FR
rs1799724	C/T	TNF	tumor necrosis factor	5'-FR
rs1799964	T/C	TNF	tumor necrosis factor	5'-FR
rs1800629	G/A	TNF	tumor necrosis factor	5'-FR
rs361525	G/A	TNF	tumor necrosis factor	5'-FR
rs4149570	G/T	TNFRSF1A	tumor necrosis factor receptor superfamily, member 1A	5'-FR
rs4149584	G/A	TNFRSF1A	tumor necrosis factor receptor superfamily, member 1A	Arg121Gln
rs1061622	T/G	TNFRSF1B	tumor necrosis factor receptor superfamily, member 1B	Met196Arg
rs5746026	G/A	TNFRSF1B	tumor necrosis factor receptor superfamily, member 1B	Glu232Lys
rs2297518	G/A	NOS2	nitric oxide synthase 2, inducible	Leu608Ser
rs1799983	G/T	NOS3	nitric oxide synthase 3 (endothelial cell)	Glu298Asp
rs20417	G/C	PTGS2	prostaglandin-endoperoxide synthase 2	5'-FR
rs5275	T/C	PTGS2	prostaglandin-endoperoxide synthase 2	3'-UTR
rs689470	C/T	PTGS2	prostaglandin-endoperoxide synthase 2	3'-UTR

*FR: flanking region, UTR: untranslated region.

### Statistical Analysis

Time to event (grade ≥2 pneumonitis or esophagitis) was based on the duration from start of radiation treatment to occurrence of toxicity. Three-year survival was also defined as the time from start of radiation treatment to the date of death or the date of last follow-up during the three year period. Hazard ratios (HRs) and 95% confidence intervals (95% CIs) for each individual SNP and endpoint combination were estimated by fitting the Cox proportional hazard model while adjusting for age, gender, clinical stage, pack years of smoking, pre-treatment performance status, treatment regimen (radiotherapy or chemoradiotherapy), radiation type, and radiation dosage. Kaplan-Meier curves and log-rank tests were used to assess differences in time to event and overall survival rates. Combined effects of unfavorable genotypes were based on the main effect analysis of individual SNPs and included those with significant (*P*<0.05) and borderline significant (*P*<0.10) associations. STATA software (version 10, STATA Corp., College Station, TX) was used for statistical analyses. Q-values were calculated to control for multiple comparisons based on an FDR value of 10% [47].
